# Editorial for the Special Issue on Piezoelectric Transducers: Materials, Devices and Applications, Volume III

**DOI:** 10.3390/mi14101862

**Published:** 2023-09-28

**Authors:** Jose Luis Sanchez-Rojas

**Affiliations:** Microsystems, Actuators and Sensors Lab, Institute of Nanotechnology, Universidad de Castilla-La Mancha, 45071 Toledo, Spain; joseluis.saldavero@uclm.es

This is the third volume of a Special Issue focused on piezoelectric transducers, covering a wide range of topics, including the design, fabrication, characterization, packaging and system integration or final applications of mili/micro/nano-electro-mechanical system-based transducers featuring piezoelectric materials and devices. The articles in this issue highlight developments in the downsizing of sensors, actuators and smart systems that are attracting significant industrial attention and have a wide range of commercially accessible transducers or a high potential to influence emerging markets [1,2,3]. With the potential for manufacturing using cutting-edge silicon integrated circuit technology or alternative additive techniques from the mili- to the micro-scale, it is now possible to replace existing products based on bulk materials in fields such as the automotive, environmental, food, robotics, medicine, biotechnology, communications, internet of things and related sectors with products having a reduced size, lower cost and higher performance [4,5,6,7,8,9,10,11,12,13,14,15,16].

This new volume comprises a total of 13 papers which highlight the latest advances in various areas in which these types of transducers are used. For instance, in reference [17], the authors effectively solved the problem of the low compensation accuracy of the nonlinear start-up error characteristics of a piezoelectric actuator under open-loop control of nanopositioning stages. Additionally, a contribution about piezocomposites for ultrasonic transducers is included in this Special Issue, providing an effective strategy for the collaborative optimization of the bandwidth and sensitivity of transducers, further guiding the design of high-performance ultrasonic transducers used in medical diagnosis [18]. The determination of the piezoelectric coefficients of MEMS devices was studied in [19], which describes a method for the characterization of piezoelectric films supporting the design and simulation of ScAlN-based piezoelectric MEMS devices with enhanced electromechanical properties.

Also included herein is a research paper about out-of-plane piezoelectric MEMS actuators equipped with a capacitive sensing mechanism to track its displacement, where the measured capacitance shows a linear relationship with the displacement [20]. Rotary and linear ultrasonic motors are also covered in various papers, which discuss, for example, the design, fabrication and characterization of cooperative microactuators featuring hybrid planar conveyance systems based on piezoelectric MEMS resonators with attached 3D-printed legs, demonstrating their application as fast, low-energy conveyors for reconfigurable electronics [21], or a new type of hybrid drive motor combining the characteristics of electromagnetic drive and piezoelectric drive devices [22,23].

In the field of miniature robot locomotion, an autonomous system with two piezoelectric plates vibrating in their first extensional mode and with attached inclined legs showed the ability to follow a pre-programmed trajectory with high precision [24]. Energy-scavenging structures are discussed in two contributions, one of which analyzes a piezoelectric heterostructure employing magnetic springs for harvesting mechanical energy from human foot strikes [25] and the other of which examines a dual-frequency vibration-based energy harvester based on coupled resonators [26]. Transducers oriented towards sensor development are also considered in this Special Issue, with demonstrations of polymeric tactile sensors being effectively used for hardness differentiation during the palpation process [27] and a polyimide-based film bulk acoustic resonator used as humidity sensor [28]. Finally, a review paper on flexible ultrasonic transducers presented recent advances in their development and practical applications in imaging systems [29].

I would like to take this opportunity to thank all the authors for submitting their papers to this Special Issue. I also want to thank all the reviewers for their efforts and time spent improving the quality of the submitted papers.

In view of the success of this Special Issue in terms of the number and quality of papers published, we plan to open a fourth volume, where we hope to continue to highlight the latest advances in piezoelectric transducers and their trend towards miniaturization, efficiency and new applications.
